# Overview of the ASM-IISc Symposium on the One Health Approach to AMR in Bengaluru, India

**DOI:** 10.1128/msphere.00211-26

**Published:** 2026-08-21

**Authors:** Amitesh Anand, Nishad Matange, Samay Pande, Vasundhra Bhandari

**Affiliations:** 1 The Department of Biological Sciences, Tata Institute of Fundamental Research 624969, Mumbai, India; 2 The Department of Biology, Indian Institute of Science Education and Research 193158, Pune, India; 3 Bacterial Ecology and Evolution Lab, Department of Microbiology and Cell Biology, Indian Institute of Science 200195 , Bengaluru, India; 4 The Department of Pharmacoinformatics, National Institute of Pharmaceutical Education and Research 231944, Hyderabad, India; Virginia-Maryland College of Veterinary Medicine, Blacksburg, Virginia, USA

**Keywords:** antibiotic resistance, drug resistance evolution, resistance surveillance

## Abstract

Antimicrobial resistance (AMR) is a systemic global threat, frequently described as a silent pandemic due to the challenges in attributing mortality to drug-resistant infections. Addressing this crisis necessitates a paradigm shift from viewing AMR as a linear consequence of antibiotic exposure to understanding it as an emergent property of ecological systems and metabolic plasticity. The ASM-IISc Symposium on the One Health Approach to AMR brought together a multidisciplinary cohort to deliberate on the drivers and solutions for resistance at the intersection of human, animal, and environmental health. Key thematic deliberations highlighted the role of technology in predicting resistance evolution and identifying novel targets for drug combinations. Furthermore, the symposium underscored the necessity of ecology-driven surveillance, extending genomic monitoring to environmental reservoirs. Ultimately, these findings establish a roadmap toward proactive, predictive, and integrated One Health strategies. Solutions demand mechanistic research and ecosystem-level interventions that bridge molecular insight with global public health strategy.

## INTRODUCTION

Amidst the sprawling campus of the Indian Institute of Science (IISc), Bengaluru, ASM hosted its first ASM Global Research Symposium in India between 29 and 31 October 2025. This symposium brought together researchers from around the world to discuss and deliberate over the many facets of antimicrobial resistance (AMR). AMR is frequently described as a silent pandemic, as it is often difficult to ascribe mortality directly to a drug-resistant infection. Yet, estimates from around the world show that the burden of AMR may already be very high. Infections due to resistant bacterial pathogens, for instance, claimed close to 5 million lives in 2019, and the number may reach 10 million deaths annually by the year 2050 (1).

The inherent difficulty in addressing this crisis stems from its multifaceted nature, as the challenge of dealing with AMR lies in the deeply cross-sectional nature of this problem. Tackling the AMR crisis requires interventions from multiple disciplines such as pharmacology, medicinal chemistry, microbiology, public health, and policy. It was also highlighted that AMR stems not solely from antibiotic misuse but also from the interplay of interconnected ecological systems and adaptive microbial processes. Chains of transmission of resistant pathogens that traverse human, animal, and environmental reservoirs amplify the AMR problem. Consequently, the ASM-IISc Symposium was appropriately focused on understanding AMR from a “One Health” perspective.

In line with this holistic approach, the symposium brought together researchers, clinicians, ecologists, and technologists to discuss the emergence, drivers, and solutions for AMR at the intersection of human, animal, and environmental health. The event featured seminars by chemists, physicians, microbiologists, epidemiologists, structural biologists, and computational biologists. Talks spanned wastewater surveys, zoonotic pathogen surveillance, metabolic pathways of bacterial tolerance, ecological drivers of resistance in natural settings, and novel diagnostic and therapeutic strategies. It also brought together researchers and policy experts from governmental and non-governmental organizations, including industries. Together, the presentations underscored that AMR is not just a clinical crisis, but a dynamic ecological and evolutionary phenomenon shaped by microbial interactions, host environments, and human activities.

To open the proceedings and define the scope of these challenges, Dr. Stefano Bertuzzi (chief executive officer, ASM) opened the meeting with his view of the key challenges in dealing with AMR. The role of animal-to-human transmissions, coupled with current limitations of drug discovery, such as specificity and rapid adaptive evolution among pathogens, were some of the key scientific challenges highlighted. However, the role of economic and environmental contributors was also underlined. For instance, while antibiotic stewardship is essential for preventing AMR, reduced use of antimicrobials means poorer returns on investment for pharmaceutical companies, disincentivizing innovation in the AMR sphere. A changing climate results in changing vector ranges that are likely to affect disease distributions in the future, as well as AMR prevalences. At the same time, a growing demand for protein in diets around the world is likely to increase the chances of zoonoses and result in many new epidemics.

Dr. Bertuzzi contextualized ASM’s efforts in this regard by briefly touching on the society’s various programs, including but not limited to its role as a scientific publisher, along different fronts that address AMR. This broad-strokes overview set the stage for a meeting where diverse issues were discussed on the same platform by researchers with different expertise. These discussions were rich, nuanced, and multidisciplinary. A few key themes emerged over 3 days of intense scientific sessions. These themes are likely to serve as a roadmap going ahead that will give direction to consolidated pan-global efforts in the AMR research and innovation sphere.

## THEME 1: THE ROLE OF TECHNOLOGY

One of the most promising avenues for addressing these systemic challenges is the application of advanced computational tools. In the age of large data and ever-new methods to handle and make sense of it, artificial intelligence (AI) is now an everyday reality. A key theme from the symposium explored how AI might serve to make better sense of surveillance data and also generate new knowledge and evidence-based hypotheses. As surveillance efforts come to the fore, the role of AI and machine learning in integrating patterns will play an indispensable role. Efforts in this direction need to, therefore, not only analyze existing data but also inform data collection strategies going ahead.

This integration of data and predictive modeling was very well exemplified by the AMRSense initiative by Dr. Tavpritesh Sethi. This platform, he explained, uses data from a number of sources and integrates antibiotic consumption, sales, and resistance rates to explain current patterns as well as predict future trends. The platform also attempts to increase public awareness using gamification. Dr. Sethi’s group is now actively working on an allied system, “AMROrbit,” that identifies country-wise risk levels for specific antibiotic–pathogen pairs. Efforts such as these are likely to play an important role in policy building and also test the efficacy of policy interventions.

Beyond its role in monitoring, another key area that AI may contribute to is in innovative drug discovery. Dr. Cesar de la Fuente’s work on AI-based mining of genomes of extant and extinct organisms for novel antimicrobial peptides was an excellent example of the power of AI (2). Work from his group has identified a number of potential candidate antimicrobial peptides from the genomes of Neanderthals, Bacteria, and Archaea. The power of computational prediction is that many of the limitations of “dirty” experimental screening can be avoided, and only a subset of promising molecules can be focused on. Indeed, his group showed close to 1 million new potential antimicrobial peptides from microbes that may be able to give us a competitive edge in the drug–bug race. The sequences of these and other potential peptides are available in “AMPSphere,” a freely accessible data resource.

These predictive capabilities are further enhanced by systemic modeling, such as the strategies for reversing antibiotic resistance from systems modeling discussed by Dr. Nagasuma Chandra from the IISc. For example, in a notable collaboration with Dr. Dipshikha Chakravortty from IISc, her lab used metabolic network modeling to identify the enzyme serine hydroxymethyltransferase (GlyA), which, when inhibited or deleted, restores (rescues) sensitivity to the antibiotic trimethoprim in resistant *Escherichia coli* (3). Similarly, in collaboration with Dr. Amit Singh (IISc), her lab has applied network-mining algorithms to genome-scale response data to uncover vulnerabilities in drug-resistant mycobacteria, identifying specific compounds that block antioxidant responses and restore antibiotic efficacy (4). These studies demonstrate that computational modeling can move beyond just describing resistance to actively identifying novel targets for drug combinations that can “reset” a pathogen’s drug sensitivity.

This technological shift also extends to the genomic analysis of diverse pathogens, as highlighted by Dr. Christina Cuomo from the Broad Institute of MIT and Harvard University and editor in chief of *Microbiology Spectrum*, who highlighted the contribution of fungal pathogens to the AMR burden. Her group is focused on understanding the structure of the global fungal outbreak and how resistance against multiple antifungals has emerged. They are using genomic approaches to delve into this issue, and this work largely emphasized the resistance mechanism associated with various classes of antifungals in *C. auris* with the help of genomic tools. Furthermore, Dr. Cuomo’s group monitored the *in vitro* stepwise progression of multidrug resistance (MDR) in *Candida auris* and reported that they observed rapid evolution of the MDR phenotype, with novel mutations in known resistance genes. For example, identification of mutations in the sterol-methyltransferase gene ERG6 linked to amphotericin B resistance is a significant step in understanding these mechanisms (5).

Complementing these findings, Dr. Sulagna Basu from the ICMR-National Institute for Research in Bacterial Infections highlighted the paradox of CRISPR-Cas and AMR. They analyzed genomes for CRISPR-Cas systems, spacers, and ARGs in *K. pneumoniae* isolates. In comparison to non-MDR strains, a lower spacer count was observed in MDR strains, indicating that greater spacer diversity in non-MDR strains suggests putative CRISPR-Cas-mediated activity (6).

## THEME 2: ECOLOGY-DRIVEN SURVEILLANCE AND CROSS-SECTOR PATHOGEN MAPPING

While technology provides the tools for analysis, the One Health approach requires extending these networks beyond hospitals and integrating information from environmental reservoirs. A core theme across sessions was the need to understand AMR and zoonotic diseases through ecological systems rather than isolated clinical frameworks. Questions of where surveillance can be most effectively and sustainably performed were central in this regard.

As a primary candidate for this expanded monitoring, wastewater and sewage are strong candidates for this purpose, and efforts in this direction are already ongoing. Dr. Farah Ishtiaq emphasized the success of wastewater-based epidemiology as a tool for monitoring, especially in resource-poor contexts. As a principal scientist at the Tata Institute for Genetics and Society, she demonstrated wastewater-based epidemiology as a scalable tool for detecting pathogens and antibiotic resistance genes (ARGs) in urban water systems in Bengaluru, revealing persistent tetracycline resistance likely driven by agricultural usage. The simplicity of this approach is particularly valuable, as the analysis of whole-genome sequencing from sewage inlets and outlets can be used to predict trends and locate potential disease hotspots. Such comprehensive analysis of wastewater surveillance for ARGs in Bengaluru city may serve as an early warning system for outbreak preparedness.

This environmental focus is being mirrored in other regions and through different vectors; Dr. Ramanan Laxminarayan of the One Health Trust pitched airports as additional sites for surveillance to understand the global circulation of resistant microbes. Furthermore, surveillance at sites of antibiotic consumption other than hospitals, such as poultry or fish farms, is needed to provide crucial data bridging information between humans and their abiotic environment (7).

Understanding the movement of pathogens between sectors is equally vital to this mapping. Dr. Nagendra Hegde, whose expertise lies in animal virology and immunology at the National Institute of Animal Biotechnology, focused on nationwide pathogen prevalence mapping spanning over 43,000 animal samples. He introduced the concept of “spillover,” i.e., the transmission of a pathogen from animal to human. From a One Health point of view, understanding what drives spillover is essential to accurately estimate the prevalence of zoonotic pathogens. This perspective was expanded by talks by Dr. Ayush Kumar (University of Manitoba) and ecologist Dr. Abi Vanak (Ashoka Trust for Research in Ecology and the Environment), who extended this view to environmental reservoirs, including soil, livestock, ticks, and wildlife. Dr. Kumar’s work focused on *Acinetobacter* strains of non-clinical origin (*A. courvalinii* and *A. seifertii*), which showed resistance patterns similar to clinical isolates, a matter of grave concern. Dr. Vanak’s talk highlighted how ticks contribute significantly to the burden of disease in cattle.

Complementing these large-scale mapping efforts, mechanistic research is revealing how resistance emerges in the wild. Dr. Samay Pande (IISc) showed that AMR maintenance can occur even in the absence of antibiotic exposure. This is critical to understanding the evolutionary mechanisms of resistance that were present in bacteria long before the discovery of antibiotics (8). Dr. Pande’s talk focused on how microbial interactions in soil communities, specifically involving the predatory bacterium *Myxococcus xanthus*, influence antibiotic resistance. His group experimentally showed that when *M. xanthus* cells die, they release molecules that create a toxic environment, selectively favoring pre-existing resistant strains (8, 9). Finally, Dr. Laasya Samhita discussed how non-genetic variations, such as mistranslation during protein synthesis, contribute to resistance (10). Her group showed that environmental stressors leading to mistranslation can make bacteria temporarily more resistant, giving them time to acquire stable genetic mutations. Collectively, these sessions highlighted that surveillance systems must integrate genomic data across cities, farms, wildlife, and clinical centers to predict future outbreaks.

## THEME 3: HOST-DIRECTED THERAPIES, MECHANISTIC BASIS OF RESISTANCE, AND METABOLIC ADAPTATION

Moving from the environment back to the host, researchers are exploring strategies that target the host’s response rather than the pathogen itself. While novel antimicrobials are typically thought of as being directed against pathogens, an alternative strategy may be to direct therapies toward the host. Host-directed therapies (HDTs) function by tweaking the immune system to prevent pathogen proliferation or facilitate clearance. One of the main perceived advantages of HDTs is likely to be a lower rate of resistance in pathogens.

Several examples of HDTs at various stages of development were discussed to illustrate this paradigm. For instance, Dr. Vasundhara Bhandari (National Institute of Pharmaceutical Education and Research) described their efforts in using vorinostat (SAHA) to manipulate histone modifications in host cells that drove more effective clearance of intracellular *Acinetobacter baumannii* (11). Importantly, this bacterial pathogen is notorious for being multidrug resistant and is therefore an ideal candidate for the design of HDTs. The success of such therapies often depends on a deep understanding of metabolic crosstalk, a concept explored in detail regarding tuberculosis.

Dr. Amit Singh (IISc), one of the leading scientists in understanding the basis of persistence in *Mycobacterium tuberculosis* (Mtb), demonstrated that Mtb survival depends on redox state, central carbon metabolism, ROS modulation, and ATP fluxes. These metabolic shifts enable antibiotic tolerance prior to heritable resistance, suggesting that targeting host–pathogen metabolic crosstalk could shorten tuberculosis therapy (12). Dr. Singh’s work highlighted that the presence of heterogeneity in the host macrophage population can delay bacterial killing during TB treatment. This was demonstrated using a redox biosensor, Mrx1-roGFP, by which they identified basal, reduced, and oxidized Mtb subpopulations, each responding differently to stress and drugs. They also showed that macrophages hosting oxidized versus reduced Mtb had distinct metabolic profiles, with oxidized ones producing more ROS and being more drug sensitive. Overall, the study highlighted how targeting metabolic and redox heterogeneity in both the host and Mtb could help improve TB treatment outcomes.

This focus on metabolic adaptation is a recurring theme in understanding clinical persistence. Another senior scientist, Dr. Raju Mukherjee (IISER Tirupati), focused on how metabolic changes in Mtb affect pathogenic outcomes. His data revealed that central carbon metabolism and antioxidant defenses ramp up when antibiotics are added, and fluxomics showed that cells push carbon away from the TCA cycle to limit ROS production. Antibiotic exposure also triggers a time- and dose-dependent ATP burst, and using protonophores to suppress this burst makes cells more tolerant to drugs like streptomycin (13). Overall, both these talks highlight that understanding the metabolism of both host and pathogen holds the key to combating antimicrobial resistance.

Beyond direct host–pathogen interaction, the broader microbial environment also plays a role in these adaptations. Though not directly an HDT, microbiome manipulation and restoration to treat resistant infections was yet another recurrent and provocative idea that emerged from the scientific talks. Dr. Colleen Kraft provided an overview of microbiome research in the context of AMR. Comparing the human gut to a forest rather than a garden, she drew parallels between the ecology of forests to apply principles of species richness and abundance to tackle the AMR problem.

These ecological principles are further supported by structural insights into the molecular systems that enable resistance. Several talks examined molecular systems that enable antimicrobial resistance through diverse mechanisms. Dr. Cesar Arias, from Houston Methodist Hospital and editor in chief of *Antimicrobial Agents and Chemotherapy*, demonstrated how daptomycin resistance arises through cell envelope remodeling mediated by the LiaFSR system, which also introduces collateral sensitivity trade-offs to other antibiotics (14). Dr. Aravind Penmatsa (IISc) presented structural insights into proton-dependent efflux pumps such as QacA, where cryo-EM analysis revealed a targetable extracellular gating loop that could guide efflux pump inhibitors (14,15).

Furthermore, regulatory shifts can drive resistance through unexpected pathways. Dr. Nishad Matange (IISER Pune) showed that resistance can be driven by increased gene expression demand, where loss-of-function mutations in mgrB activate the PhoPQ system, ultimately elevating DHFR levels and promoting trimethoprim resistance (16). Complementing these findings, Dr. Anil Kumar Singh (CSIR-North East Institute of Science and Technology) demonstrated that plasmid-borne β-lactamases in *Enterobacteriaceae* spread efficiently due to high conjugation frequencies and low fitness costs, allowing resistance traits to persist even in the absence of antibiotic pressure (17). Together, these studies highlight how envelope remodeling, efflux regulation, gene-expression shifts, and plasmid transmission collectively shape the evolution of AMR. These results establish that resistance is driven not only by mutations but also by regulatory rewiring, metabolic state, and environmental stress, creating windows of tolerance that permit the evolution of stable AMR genotypes.

## THEME 4: EMERGING DIAGNOSTIC AND THERAPEUTIC SOLUTIONS

The translation of these mechanistic insights into the clinic requires innovative diagnostic and therapeutic platforms that move beyond traditional antibiotic discovery. Several speakers presented strategies for such advancements, such as Dr. Reety Arora, senior scientist and head of R&D at CRISPRBITS, who showcased CRISPR-based platforms capable of rapidly detecting resistance plasmids in mixed microbial cultures. She introduced EdiCrisp, a CRISPR-based tool for bacterial genome editing, and PathCrisp, a Cas12-driven diagnostic platform that uses trans-cleavage activity to provide a fluorescence-based readout. Their success highlights the phenomenal rise of startups supported by C-CAMP.

In terms of new chemical entities, researchers are developing hybrid molecules to bypass existing resistance pathways. Dr. Shashikala Verma (Banaras Hindu University) reported that hybrid ciprofloxacin–naphthalimide derivatives can lower MIC values by nearly 10-fold against resistant *Staphylococcus aureus*. Her work involved creating ciprofloxacin–naphthalimide conjugates using either cleavable or non-cleavable linkers of different lengths and side-chain groups. These conjugates were effective across both sensitive and resistant strains of gram-positive and gram-negative bacteria, suggesting they may act through mechanisms that bypass common resistance pathways. Overall, the work showed how hybrid molecules could offer a promising direction for developing next-generation antibacterial agents.

Innovative clinical solutions are also being tailored for specific regional and anatomical needs. Dr. Devarshi Gajjar, a professor from Maharaja Sayajirao University of Baroda, highlighted how the H58 (4.3.1) lineage of *Salmonella* Typhi has expanded into region-specific sublineages, suggesting β-lactam/β-lactamase inhibitor combinations as effective alternatives for managing XDR *Salmonella* Typhi (18). Similarly, Dr. Ranjana Pathania (Indian Institute of Technology Roorkee) presented the design of phenylboronic acid (PBA) derivatives as non-β-lactam β-lactamase inhibitors. These PBA derivatives effectively restore carbapenem activity in KPC-2-producing isolates and significantly reduce bacterial burden in a murine pneumonia model, highlighting their promise as adjunct therapeutics (19).

Anatomical specificities also drive the need for new delivery systems, particularly in ophthalmology. In the clinical sphere, Dr. Prashant Garg from the L V Prasad Eye Institute emphasized the alarming issues of AMR in ocular infections leading to vision loss. There is a dire need for new approaches, such as better formulations to enhance drug bioavailability and sustained-release solutions like conjunctival patches, microneedle patches, or injectable implants. Complementing this, Dr. Radha Rangarajan, director of CSIR-CDRI-Lucknow, highlighted the hurdles in drug discovery and advocated for combination therapies that target more than one bacterial pathway to prevent resistance generation.

Despite the hurdles in the global pipeline, recent progress has been made with several first-in-class approvals. Dr. Helen Boucher from Tufts University School of Medicine presented new agents being developed to treat resistant infections, noting that while no antibiotics were approved by the FDA from 2020 to 2023, sulbactam–durlobactam was approved in May 2023 for carbapenem-resistant *Acinetobacter baumannii*. Other new approvals include sulopenem and pivmecillinam for uncomplicated UTIs and gepotidacin mesylate, a first-in-class triazaacenaphthylene antibiotic. India has also made notable contributions to this global pipeline. India’s contributions include EXBLIFEP, the first antibiotic developed in India, approved in 2024, along with promising pipelines from Bug Works and Wockhardt.

Finally, speeding up the clinical response is dependent on modernizing diagnostic speeds. Dr. Romney Humphries from Vanderbilt University Medical Center and editor in chief of *Journal of Clinical Microbiology* discussed modernizing antimicrobial susceptibility testing methods to include both phenotype and genotype assessment. Technologies such as rapid multiplex PCR and next-generation sequencing are now available to provide blood culture diagnosis and susceptibility results in just 2–4 hours, significantly reducing the testing time compared to traditional methods.

## THEME 5: FIGHTING MULTIDRUG RESISTANCE

The final sessions focused on the most acute aspect of the crisis: the burning issue of MDR. The focus here was to understand resistance mechanisms and therapeutic strategies to combat these resilient pathogens. For example, Dr. Bhabatosh Das (BRIC-Translational Health Science and Technology Institute) discussed his recent work on alternatives to antibiotics. His team decoded more than 3,000 bacterial genomes and observed shared AMR traits among clinically relevant pathogens. To gain insights into resistance heterogeneity, they developed a robust, genetically engineered high-throughput screening platform based on *Vibrio cholerae* and *Escherichia coli* reporter strains. From a 4,417-molecule screen, they found over 41 resistance-inhibiting compounds, including BMM_1567, which potentiates aminoglycoside activity against MDR ESKAPE pathogens. In a murine abscess model, BMM_1567 combined with spectinomycin significantly reduced bacterial burden and inflammation without detectable resistance development.

Multidrug resistance is not limited to bacterial threats; it is an escalating concern in the fungal kingdom as well. Dr. Sanchita Mitra (L V Prasad Eye Institute) focused on emerging antifungal resistance (AFR), noting there are 150 million cases per year accounting for 1.7 million deaths. The widespread use of azole fungicides in agriculture has contributed to the emergence of azole resistance in clinical settings. Alarmingly, plant and animal fungal pathogens are increasingly implicated in invasive human infections, yet remain poorly studied. Strategies to slow AFR include new antifungal discovery, combination therapy, stewardship policy, and early diagnostics. This was echoed by Dr. Anuradha Chowdhary, who emphasized the role of reference laboratories in identifying emerging resistant fungal pathogens.

Unexpected environmental and lifestyle factors are also being explored for their potential to either exacerbate or mitigate resistance. Dr. David Scott (University of Louisville) discussed the impact of marijuana usage on AMR, noting that while cannabidiol (CBD) is a marijuana-derived antibacterial agent, various oral *Treponema* spp. have shown resistance to it. However, synergy was observed between CBD and LL37 (cathelicidin) mimicking antimicrobial peptoids against *Treponema denticola*. Additionally, Dr. Neta Sal-Man (Ben-Gurion University) introduced research on how diet affects the virulence of enterohemorrhagic *Escherichia coli* (EHEC). Her team showed that casein-derived peptone from milk protein suppresses EHEC’s primary virulence mechanism, suggesting its potential as a promising antivirulence therapeutic.

## CROSS-CUTTING DISCUSSIONS AND PERSPECTIVES

Beyond the individual presentations, several broader themes emerged repeatedly during discussions throughout the symposium. A recurring point was that antimicrobial resistance can no longer be viewed solely through the lens of antibiotic use and misuse. Rather, resistance emerges from a complex interplay between microbial evolution, ecological interactions, host biology, environmental reservoirs, and human activities. This perspective resonated strongly with the One Health framework that underpinned the meeting.

Participants also highlighted the need to move beyond reactive approaches to AMR management. Advances in artificial intelligence, systems biology, genomics, and environmental surveillance are creating opportunities to anticipate resistance emergence before it becomes clinically problematic. At the same time, several discussions emphasized that technological advances alone will be insufficient without coordinated efforts to integrate data, expertise, and interventions across clinical, agricultural, veterinary, and environmental settings.

Another recurring theme was the importance of broadening the search for solutions. For this, many speakers and audience members highlighted complementary approaches, including host-directed therapies, combination treatments, microbiome-based interventions, antivirulence strategies, and improved diagnostics. Together, these discussions reinforced the view that durable solutions to AMR will require a portfolio of approaches rather than reliance on any single intervention. Taken together, the discussions underscored a shift in thinking that was evident throughout the symposium; that is, antimicrobial resistance is best understood as an ecological and evolutionary challenge with consequences for human, animal, and environmental health. Addressing it will therefore require equally integrated scientific and societal responses.

## CONCLUSIONS, FUTURE DIRECTIONS, AND POLICY RECOMMENDATIONS

Addressing antimicrobial resistance requires coordinated scientific, public health, and technological initiatives that bridge molecular insight with global strategy. Scientifically, priorities should include building multiomics models to predict resistance evolution from metabolic states, expanding structural studies of efflux pumps, and targeting tolerance mechanisms, such as redox balance and ATP flux, as adjunct therapies. On the public health and policy front, progress depends on establishing integrated genomic surveillance networks across wastewater, livestock, and hospital systems, alongside stricter regulation of agricultural antibiotic usage. Translational efforts must focus on deploying CRISPR-based point-of-care diagnostics, validating AI-designed antimicrobial peptides, and implementing standardized cross-sector data frameworks. Ultimately, the symposium underscored a paradigm shift: AMR is an emergent property of ecological systems and metabolic plasticity rather than a simple consequence of antibiotic exposure. By adopting these proactive and integrated One Health approaches, the global community can establish a roadmap to mitigate the AMR crisis.
